# Potential impact of co-payment at point of care to influence emergency department utilization

**DOI:** 10.7717/peerj.1544

**Published:** 2016-01-21

**Authors:** Zachary Baum, Michael R. Simmons, Jose H. Guardiola, Cynthia Smith, Lynn Carrasco, Joann Ha, Peter Richman

**Affiliations:** 1 Department of Emergency Medicine, Christus Spohn/Texas A & M Health Science Center, Corpus Christi, TX, United States; 2 Department of Mathematics, Texas A & M University—Corpus Christi, Corpus Christi, TX, United States; 3 Graduate Medical Education, Christus Spohn/Texas A & M Health Science Center, Corpus Christi, TX, United States

**Keywords:** Emergency department, Co-payment, Utilization

## Abstract

**Background.** Many proponents for healthcare reform suggest increased cost-sharing by patients as a method to reduce overall expenditures. Prior studies on the effects of co-payments for ED visits have generally not been directed toward understanding patient attitudes/behavior at point of care.

**Objectives.** We conducted a survey at point of care to test our hypothesis that a significant number of patients with urgent chief complaints might have avoided the ED if asked to provide a co-payment.

**Methods.** Cross-sectional study design. Stable, oriented, consenting patients at an inner-city, academic ED were consecutively enrolled at hours in which trained research associates were available to assist with data collection. Enrolled patients completed a written survey providing demographic/chief complaint information, and then were asked whether 13 interval amounts of co-payment ranging from 0 to >500 would have impacted their decision to visit the ED. Categorical data are presented as frequency of occurrence and analyzed by chi-square; continuous data presented as means ± standard deviation, analyzed by *t*-tests. ORs and 95% confidence intervals provided. Primary outcome parameter was the % of patients who would have avoided the ED if asked to pay any co-payment for several urgent chief complaints: chest pain, SOB, and abdominal pain.

**Results.** A total of 581 patients were enrolled; 63.1% female, mean age 42.4 ± 15.1 years, 65% Hispanic, 71.2% income less than 20,000, 28.6% less than high school graduate, 81.3% had primary care physician, 57.6% had 2 or more ED visits/past year. Overall, 30.2% of patients chose 0 as the maximum they would have been willing to pay if it was required to be seen in the ED. 16/58 (28%; 95% CI [18–40%]) of chest pain patients, 9/43 (20.9%; 95% CI [11–35%]) of SOB patients, and 24/127 (26.8%; 95% CI [13–27%]) of abdominal pain patients would have been unwilling to pay a co-pay. Patients with income >20,000 were more willing to pay a co-payment (OR = 2.55; 95% CI [1.59–4.10]). No significant relationship was identified between willingness to pay for: gender, race, education, established primary care provider, and frequency of ED visits.

**Conclusion.** Overall, 30.2% of our patients would not have accepted a co-pay in order to be seen, including more than 20% of the patients with chest pain, shortness of breath, and abdominal pain respectively.

## Introduction

The cost of healthcare has been a growing concern for many decades in our country. During 2011, CMS estimated health care expenditures in the US at $2.7 trillion overall and $8,680 per person (Centers for Disease Control Prevention, 2010). Emergency treatment is a growing component of health care spending with nearly 130 million annual emergency department (ED) visits. Prior investigation suggests that as many as half of these visits are “unnecessary,” representing $38 billion in annual spending that is potentially avoidable (National Health Expenditures, 2011).

Proponents for healthcare reform often suggest that patients should increasingly cost-share out-of-pocket as a method to reduce overall expenditures. There have been numerous studies in which investigators have attempted to evaluate the effect of cost sharing on the medical system, and they have found variable results in terms of its impact on patient health resource utilization (Becker et al., 2013; Chernew et al., 2008; DeVries, Li & Oza, 2013; Flores-Mateo et al., 2012; Hsu et al., 2006; Keeler & Rolph, 1983; Magid et al., 1997; Newhouse et al., 1981; O’Grady et al., 1985; Reed et al., 2005; Selby, Fireman & Swain, 1996; Shapiro et al., 1989; Shapiro, Ware & Sherbourne, 1986; Wharam et al., 2007; Wharam et al., 2013; Wong et al., 2001). Several investigations have found reductions in ED use following the introduction of a co-payment (Hsu et al., 2006; Keeler & Rolph, 1983; O’Grady et al., 1985; Reed et al., 2005; Selby, Fireman & Swain, 1996; Shapiro, Ware & Sherbourne, 1986; Tzeel & Brown, 0000). Meanwhile, other studies have raised concerns that such payments cause risk of disparities for patients of lower socioeconomic status (Chernew et al., 2008; Shapiro et al., 1989; Wharam et al., 2007; Wharam et al., 2013; Wong et al., 2001).

These prior studies are generally limited by the use of phone and medical records to retrospectively determine the urgency of an ED visit after the outcome for each patient was known. From many standpoints, it is important to understand how patients with potentially urgent chief complaints would respond to a co-payment without the benefit of hindsight. We conducted a survey at point-of-care to test our hypothesis that a significant number of patients with urgent chief complaints might avoid the ED if they were asked to provide a co-payment prior to evaluation.

## Methods

### Study design

We conducted a cross-sectional study designed to evaluate patients’ willingness to pay a co-payment in order to receive ED care.

### Setting

The study was conducted at Christus Spohn Memorial Hospital in Corpus Christi, Texas. The facility is a major teaching affiliate of Texas A & M Health Science Center, a level-two trauma center, and serves an inner-city population. The annual ED census is 45,000 patients. The Christus Spohn institutional review board reviewed the study protocol and provided a status of exempt (IRB #2014-004).

### Population

Our study included a convenience sample of medically stable, verbally consenting, adult patients age >18 years that presented to the emergency department during a two-month period. Patients were excluded for any of the following reasons: refusal to provide consent, pregnancy, and inability to complete the questionnaire due to clinical instability, severe pain, or disorientation as determined by a study physician. Patients who were initially unstable or in severe pain were eligible for inclusion once their condition was stabilized.

### Study protocol

Patients were consecutively enrolled at hours in which trained research associates (college students) were available to assist with data collection. These hours were variable and included overnight as well as weekend days. Enrolled patients completed a written survey providing demographic information including sex, age, race, income and education as well as chief complaint information, and then, were asked whether 13 interval amounts of co-payment ranging from $0 to >$500 would have impacted their decision to visit the ED.

### Statistical analysis

Categorical data are presented as frequency of occurrence and were analyzed by chi-square. Continuous data presented as means ± standard deviation and were analyzed by *t*-tests. Odds ratios and 95% confidence intervals are also provided. The primary outcome parameter was the percentage of patients who would have avoided the emergency department if asked to pay any co-payment for several urgent chief complaints: chest pain, SOB, and abdominal pain.

## Results

During the study period, 581 patients were enrolled. Table 1 summarizes the characteristics of the study group. The study group was relatively young (mean age 42.4 ± 15.1 years) and 63.1% were female. Typical of inner-city populations in the Southwestern US, 65% of the patients were Hispanic and from a generally low socioeconomic status (71.2% with income less than $20,000).

Overall, 30.2% of patients chose $0 as the maximum amount they would have been willing to pay if required by the ED in order to be seen. A relatively large percentage of patients with potentially urgent chief complaints might have avoided ED care with the requirement of a co-pay, including 16/58 (28%; 95% CI [18–40%]) of chest pain patients, 9/43 (20.9%; 95% CI [11–35%]) of patients with shortness of breath, and 24/127 (26.8%; 95% CI [13–27%]) of those with abdominal pain. Patients with income >$20,000 were more willing to pay co-payment (OR = 2.55; 95% CI [1.59–4.10]). We did not identify any significant relationship between willingness to pay a co-payment in order to receive ED treatment and the following variables: gender, race, education, established primary care provider, and frequency of ED visits.

**Table 1:** Study group characteristics (*N* = 581).

Mean age	42.4 ± 15.1 years
Female gender	63.1%
Hispanic	65%
Income ≤ $20,000	71.2%
<High school graduate education	28.6%
Established primary care physician	81.3%
2 or more ED visits past year	57.6%

## Discussion

Numerous investigators have evaluated the effect of cost sharing on the medical system as well as specifically with regard to patient utilization of the emergency department (DeVries, Li & Oza, 2013; Hsu et al., 2006; Newhouse et al., 1981; O’Grady et al., 1985; Reed et al., 2005; Selby, Fireman & Swain, 1996; Shapiro et al., 1989; Shapiro, Ware & Sherbourne, 1986; Wharam et al., 2007; Wharam et al., 2013; Wong et al., 2001; Tzeel & Brown, 0000). In most cases, reports have shown evidence of reduced ED visits when co-payments are required for a wide range of respective cohorts (Hsu et al., 2006; Keeler & Rolph, 1983; O’Grady et al., 1985; Reed et al., 2005; Selby, Fireman & Swain, 1996; Shapiro, Ware & Sherbourne, 1986; Tzeel & Brown, 0000). Hsu et al. (2006) performed a quasi-longitudinal study examining three years of patient records from a prepaid integrated health care delivery system when co-payments of $0–$100 were introduced (approximately 2.5 million patients). The authors found that ED visits were reduced throughout the range of co-payment groups. Further, they noted that co-payments did not increase ICU and hospital admissions, nor was there an increased number of patient deaths. These results confirmed the group’s preliminary findings in a smaller study during which they surveyed 932 patients by telephone within a large health care delivery system and found that patients tended to seek alternative sources of treatment rather than avoid the ED care altogether when confronted with co-payments (Reed et al., 2005).

Although the results of such studies suggest that co-payments may be imposed safely without reducing medically necessary ED visits, other authors have raised concerns that cost-sharing limits access to health care and increases disparities in outcomes for patients of lower socio-economic status (Chernew et al., 2008; Shapiro et al., 1989; Wharam et al., 2007; Wharam et al., 2013; Wong et al., 2001). Most of these prior studies were not focused specifically on ED utilization and/or were conducted several decades in the past. However, more recently, Wharam, Zhang, Landon et al. analyzed the ED visits and hospitalizations over two years among more than 60,000 enrollees insured through high deductible plans offered by small employers in the state of Massachusetts. They found that high deductible members from the lower socio-economic classes reduced high-acuity ED visits by 25–30%, while hospitalizations rose in the second year after joining the plan (Wharam et al., 2013).

The findings of our investigation, which examines the sentiment of a predominantly poor study group at point of ED care, are complementary to the observations of Wharam, Zhang, Landon et al. The study is novel for providing an opportunity for patients to report how they might respond to a co-payment within the context of their current ED visit and chief complaint, rather than by phone or examining medical records when the acuity/appropriateness of the visit is determined with the benefit of a retrospective viewpoint. Overall, within our study group, 30.2% of patients reported that they would not have been willing to pay a co-payment in order to be seen that day. This included more than 20% of each of the patient groups with the following urgent chief complaints: chest pain (28%), shortness of breath (20.9%), and abdominal pain (26.9%) respectively. Physicians, as much as patients, often struggle to identify when symptoms within these chief complaint groups represent emergent pathology. Thus, our findings suggest the potential for serious adverse events if a point-of-care co-payment was a requirement to be seen at our ED. This is especially true for our inner-city patient population with limited access to other venues for rapid health care evaluation.

## Limitations and Future Questions

Our study has several limitations that warrant discussion. The opportunity to focus on patients at point of care and within the context of their current chief complaint holds several of the aforementioned advantages over previous investigations. However, our survey instrument has not been previously validated, and we asked patients a written question regarding their ED utilization in the setting of a hypothetical co-payment. Patients’ answers may not reflect how they would actually respond if a payment was truly required. Nonetheless, before implementing such a requirement, we would argue that it is worthwhile to examine the question a hypothetical manner first so as to identify the potential risk of patients not presenting with urgent complaints. Further, we did not collect data regarding the eventual outcome of those patients that reported that they would have avoided ED care. Thus, the actual risk of seriously ill patients leaving before treatment or deciding to not come to the ED is unknown. Future studies should follow patients who might refuse ED co-payments forward from their triage through their diagnostic work up and outcomes.

We did not track patient refusals to participate, so we do not know how those who refused might have responded to the same questions and/or if those patients matched the participants in demographic and other characteristics. Furthermore, with respect to external validity, our patient population is predominantly Hispanic and relatively poor. Thus, our results may not be generalizable to other settings and populations. However, as Latinos represent the fastest growing demographic group in the US, this population certainly warrants more study as cultural and language barriers may influence their decision-making to visit the ED.

## Conclusions

Overall, 30.2% of our patients would not have accepted a co-payment in order to be seen in the ED. With respect to serious chief complaints, more than 20% of patients with chest pain (28%), shortness of breath (20.9%), and abdominal pain (26.9%) respectively reported that they would have avoid the ED under such circumstances.

## Supplemental Information

10.7717/peerj.1544/supp-1 Supplemental Information 1 Co-payment data set

## Additional Information and Declarations

### Data Availability

The following information was supplied regarding data availability: The research in this article did not generate any raw data.
